# COUP-TFII in Health and Disease

**DOI:** 10.3390/cells9010101

**Published:** 2019-12-31

**Authors:** Simone Polvani, Sara Pepe, Stefano Milani, Andrea Galli

**Affiliations:** 1 Department of Experimental and Clinical Biomedical Sciences “Mario Serio”, Gastroenterology Unit, University of Florence, viale Pieraccini 6, 50139 Firenze, Italy; simone.polvani@unifi.it (S.P.); stefano.milani@unifi.it (S.M.); 2 Department of Experimental and Clinical Medicine, University of Florence, largo Brambilla 50, 50139 Firenze, Italy; 3 Istituto per la Ricerca, la Prevenzione e la rete Oncologica (ISPRO), viale Pieraccini 6, 50139 Firenze, Italy; sarapepe93@hotmail.com; 4 Department of Medical Biotechnologies, University of Siena, via M. Bracci 16, 53100 Siena, Italy

**Keywords:** NR2F2, COUP-TFII, cancer, metabolism, NF-κB, EMT, angiogenesis, development, metabolism

## Abstract

The nuclear receptors (NRs) belong to a vast family of evolutionary conserved proteins acting as ligand-activated transcription factors. Functionally, NRs are essential in embryogenesis and organogenesis and in adulthood they are involved in almost every physiological and pathological process. Our knowledge of NRs action has greatly improved in recent years, demonstrating that both their expression and activity are tightly regulated by a network of signaling pathways, miRNA and reciprocal interactions. The Chicken Ovalbumin Upstream Promoter Transcription Factor II (COUP-TFII, NR2F2) is a NR classified as an orphan due to the lack of a known natural ligand. Although its expression peaks during development, and then decreases considerably, in adult tissues, COUP-TFII is an important regulator of differentiation and it is variably implicated in tissues homeostasis. As such, alterations of its expression or its transcriptional activity have been studied and linked to a spectrum of diseases in organs and tissues of different origins. Indeed, an altered COUP-TFII expression and activity may cause infertility, abnormality in the vascular system and metabolic diseases like diabetes. Moreover, COUP-TFII is actively investigated in cancer research but its role in tumor progression is yet to be fully understood. In this review, we summarize the current understanding of COUP-TFII in healthy and pathological conditions, proposing an updated and critical view of the many functions of this NR.

## 1. Introduction

The nuclear receptors (NRs) belong to a family of evolutionary conserved proteins acting as ligand-activated transcription factors. The defining features of NRs are two highly conserved domains: the DNA binding domain (DBD) and the ligand binding domain (LBD), separated by a hinge region. Interestingly, the DBD and LBD may function independently from one another [1,2]. The DBD domain is necessary for the binding to DNA sequences found in the promoter regions of NR target genes, called hormone response element (HRE), which are specific for each NR. HRE are short nucleotide sequences usually present in pairs that are separated by a spacer of various lengths; the pairs can be in the same orientation, as in the direct repeats (DR) HRE, or in opposed orientation, like in the inverted (IR) HRE. The LBD instead recognizes and binds the ligand, usually small hydrophobic molecules like retinoids, vitamins and lipophilic hormones, but also xenobiotics or antibiotics [3].

The ligand induces a conformational change of the NR molecule necessary to modify the binding properties of the receptors and the affinity for co-repressors or co-activators. HRE bound and unbound NRs act as monomers, homodimers or heterodimers (usually with retinoid X receptor, RXR) in the target promoter.

Initially identified in 1985 [4], the NRs are classified according to their evolutionary distance in six subfamilies, numbered from 1 to 6; each subfamily is further divided in several groups (indicated by letters, starting with A), followed by a number to indicate the specific member. A seventh family of NRs, NR0, has been introduced to identify NRs missing the DBD [1,3].

NRs are also classified according to the existence of known ligands and their mode of action and functional status before the ligand binding. Some of the NRs were isolated and characterized after the identification of their ligand (i.e., the endocrine NRs); however, roughly half of the 48 NRs have been isolated without previous knowledge of their ligand and were classified as “orphan”, meaning that a specific ligand was missing. The existence of orphan receptors had important implications for endocrinology research, paving the way towards the “reverse endocrinology” concept [5]. The “reverse endocrinology” concept represents a substantial paradigm shift in endocrinology: Before the identification of orphan receptors, new hormones were discovered and isolated, and their physiological effects studied and subsequently used to identify their receptors; conversely, in “reverse endocrinology” the ligand discovery is driven by the identification of their receptors and it often uncovered previously unknown and unexpected signaling pathways. RXR was the first of the “orphan” receptor for whom its specific natural ligand, 9-*cis*-retinoic acid (9-*cis*-RA), was identified; all orphan NRs with identified ligands are indicated as “adopted”. Adopted NRs are, for example, Farnesoid X receptor (FXR), liver X receptor (LXR), peroxisome-proliferator activated receptors (PPARs) and hepatocyte nuclear factor 4 (HNF4) [1].

The NRs mode of action (and functional status before the binding of the ligands) distinguishes NRs in four classes or types: type I NRs are complexed with heat shock proteins (HSP), reside in the cytosol before binding the ligands and work as dimers recruiting co-activators; type II receptors work as heterodimers, reside in the nucleus bound to the DNA with co-repressors in the absence of the ligands; type III NRs work essentially as homodimers; and, finally, type IV NRs are poorly studied and bind to HRE preferentially as monomers [3].

Functionally, NR activity is essential during development and the adult life and NRs are involved in almost every physiological process (e.g., cell commitment and differentiation, organ development, regulation of metabolism). Given the broad range of NR functions, it is not a surprise that their altered activity has been linked to the development of human diseases.

## 2. COUP-TFII: An Overview on Structure, Mechanism of Action and Expression

Chicken Ovalbumin Upstream Promoter Transcription Factors (COUP-TFs) are orphan NR transcription factors belonging to subfamily 2 (NR2) group F of the NR family [1,3,6].

In vertebrates, three homologous subtypes were identified: COUP-TFI (EAR-3, NR2F1), COUP-TFII (ARP-1, NR2F2) and COUP-TFIII (EAR-2, NR2F6) [7,8,9,10,11].

**Figure 1:**
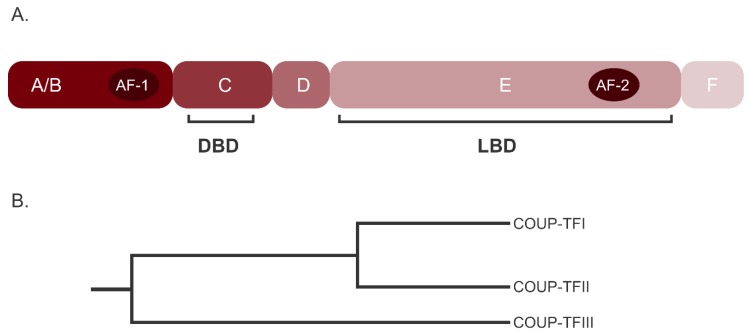
COUP-TFII protein and family relation. (**A**) The main isoforms of COUP-TF proteins possess the typical features of the NR domain structure of COUP-TFs nuclear receptor (NR). (**B**) Phylogram (guide tree) with cladogram branch length of the NR2F family showing that separation of COUP-TFIII from COUP-TFII and COUP-TFI is probably an early evolutionary event (data obtained from ClustalOmega alignment of human NR2F1, NR2F2 and NR2F6). Based on References [1,2,3].

The main human isoforms of COUP-TFI, II and III are similar in length, ranging from the 404 amino acids (AA) for COUP-TFIII to the 423 AA of COUP-TFI. The COUP-TFs proteins share the six regions structure of NRs (from N-terminal to C-terminal): the A/B region containing the activating function 1 (AF1), necessary for the recruitment of co-activators; the C region with the DBD; the hinge or D region that probably double as the nuclear localization sequence (NLS); the E region with the AF-2 and the LBD; and followed by the F or C-terminal region (Figure 1A).

COUP-TFI and II NRs have the highest homology among the COUP-TFs, especially in the DBD and LBD, whereas homology in the N-terminal is around 50% [12]. Indeed, homology is 98% in the N-terminal DBD and 96% in C-terminal LBD [10]; interestingly, homology in the N-terminal AF-1 is only around 50%. Instead, COUP-TFIII is more divergent, suggesting that the separation of COUP-TFIII from COUP-TFI/COUP-TFII is an early evolutionary event in the history of the NR2F group (Figure 1B).

### 2.1. COUP-TFI and COUP-TFII: Similarities and Differences

Both COUP-TFI and COUP-TFII show an elevated degree of evolutionary conservation and this suggests their crucial role in cellular function; moreover, their high homology could be indicative of redundancy and of overlapping functions. Nonetheless, the two receptors are in distinct loci, and regulate different physiological functions; these differences may be ascribed to differences in the regulation of gene expression associated with distinct positions in the genome and with a dissimilar affinity for co-activators and co-repressor that are recruited by AF-1, a region of low homology between the receptors.

Specifically, the *COUP-TFI* gene is located on chromosome 5 in humans and on chromosome 13 in mice [13]. It is expressed predominantly during the development of the peripheral nervous system (PNS) and the central nervous system (CNS) and it is involved in early neurogenesis [12,14]. Interestingly, *COUP-TFI* knock out (KO) is lethal in perinatal life but not during embryogenesis, with defects localized mainly in the central nervous system (CNS) [15]. Instead, the *NR2F2* gene encoding for COUP-TFII is located on chromosome 15q26 in humans and on chromosome 7 in mice [13]. The gene is organized in three exons, plus a newly identified exon in the 5′ of the putative *COUP-TFII* promoter. The NR is mainly expressed in mesenchymal cells during organogenesis and is indispensable for normal development. In fact, *COUP-TFII* KO mice show several vascular abnormalities in the heart and brain, and they die around the tenth day of embryonic life, while two-thirds of heterozygous mice die during the first week of life [16]. Despite the COUP-TFII influence in several aspects of embryogenesis, its expression declines dramatically until reaching basal levels in adult healthy tissues.

In the adult, an increase in COUP-TFII expression occurs under pathological conditions, such as cardiovascular diseases [17] or cancer, in which it regulates tumor growth and metastasis by modulating tumor angiogenesis [3,18].

The expression pattern of COUP-TFI and COUP-TFII have been thoroughly reported elsewhere (see [12,19,20,21,22] for reviews).

### 2.2. COUP-TFII Signaling: Activation, Repression and Transrepression

**Figure 2:**
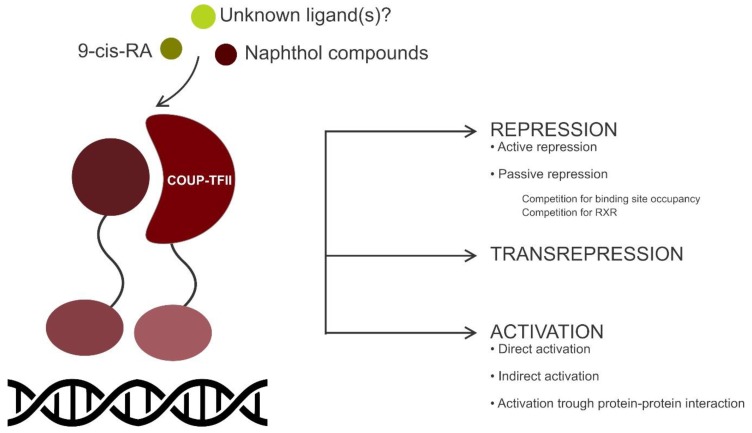
COUP-TFII dimerization and mechanism of action. COUP-TFII may form homo- and heterodimers and may act as a transcriptional repressor or activator, in a cell-dependent manner. Natural ligands for COUP-TFII are unknown, but the NR may be activated by a high concentration of 9-*cis*-RA and inhibited by naphthol compounds. Based on References [3,23,24,25,26,27,28,29,30,31,32,33].

NRs control gene transcription by one of several mechanisms. Activation of target genes can be direct (a consequence of a direct binding to HRE), indirect (when the NR acts as an accessory factor) or coming from a protein–protein interaction. Similarly, repression by NR can be direct (or active) or indirect (or passive) (e.g., competition for RXR heterodimerization) or obtained after direct binding to other NRs in a process called transrepression (Figure 2).

Even if COUP-TFs were originally identified as transcriptional activators of the chicken ovalbumin gene [34], COUP-TFII can serve as a transcriptional activator or repressor in a cell-type-dependent and gene-specific manner through binding to AGGTCA DR [23]. It has been shown to interfere with the transactivation of other nuclear hormone receptors (e.g., PPAR, VDR, TR and RAR) through the direct occupation of their DNA-binding site or the sequestration of RXR [23,24,25,26,27].

RXR is the heterodimeric partner of choice of a multitude of NRs and is able to bind to orphan NRs increasing their transactivation potential [28,35,36]. In homodimers, RXR responds exclusively to 9-*cis*-RA, but it can form a heterodimer, for example with PPARs, thus becoming able to bind PPAR-specific ligands. Although COUP-TFII may bind DNA as a homodimer, it forms stable DNA-binding heterodimers with RXR and other NR and transcription factors [24,25,29]. When sequestered by COUP-TFII, RXR is no longer available to bond with other NRs, thus interfering with the transactivation of their target genes [24,27].

Moreover, COUP-TFII can act as a transcriptional inhibitor thanks to the recruitment of co-repressors. Its interaction with the silencing mediator for retinoic acid receptor and thyroid hormone receptor (SMRT) inhibits *PPARγ* gene expression, necessary for adipocyte differentiation, in a Wnt/β-catenin pathway dependent manner [30]. In addition, COUP-TFII can also inhibit transcription by transrepression, by directly binding to the LBD of nuclear hormone receptors [31].

COUP-TFII is a positive regulator of the transcription of several genes by binding to DNA elements and directly or indirectly activating gene expression. For example, it acts synergistically with HNF4 to activate the rat cholesterol 7α-hydroxylase (*CYP7A1*), which catalyzes the first step in the bile acid synthesis pathway [37]. Moreover, COUP-TFII (physically interacting with HNF-4) can increase the activation of the *HNF-1α* gene promoter in the liver if compared to that determined by HNF-4 alone [38]. Despite these pieces of evidence of a synergistic interaction between COUP-TFII and HNF-4, it has been shown that COUP-TFII is also able to act differently by inhibiting HNF-4 transactivation according to a different promoter context [39], confirming the dual role that this receptor plays in a context-dependent manner.

### 2.3. COUP-TFII Protein Structure: Isoforms, the LBD and the Ligands

The existence of COUP-TFII isoforms has been acknowledged only recently [40,41]. While the NR primary isoform has the structure described above, at least one other isoform has been discovered in mice, while three more have been found in humans (one of these is the orthologue of the second mouse isoform, identified as variant 2, or V2). All these newly identified isoforms are shorter than the canonical COUP-TFII (COUP-TFII variant 1, COUP-TFII_V1) and they have in common a different N-terminal region that may originate from the use of the alternative first exon cited above (in mice and humans) or, in humans only, by protein translation beginning inside the second exon. All these isoforms miss the DBD but have the same LBD of the COUP-TFII_V1, casting doubts on their function as standard NRs. Indeed these new isoforms have been demonstrated to either increase or decrease canonical COUP-TFII activity in a cell-dependent manner: In hepatocellular carcinoma cells, COUP-TFII_V2 reduces the ability of COUP-TFII_V1 to activate *CYP7A1*, while in human embryonic stem cells they have been show to increase the activity of the longest isoform [40,41]; given the lack of a DBD, we may hypothesize that they might compete for the binding of co-repressors or co-activators, but how they truly act is still unknown.

In an effort to identify putative ligands for COUP-TFII, Kruse et al. [32] focused their attention on the human COUP-TFII LBD, demonstrating a peculiar auto-inhibited disposition in the crystal structure [32]. Usually, the NR activity is mainly guaranteed by the conformational states of the AF-2 helix, located in the LBD region [42]. The binding of an agonist ligand stabilizes the AF-2 in an active conformation that makes the LBD able to recruit co-activator proteins. In contrast, antagonist ligands promote an inhibited conformation of the LBD region [43]. Instead, for COUP-TFII in the absence of ligands, the auto-repressed conformation is due to the interaction between the AF-2 and the co-factor binding site, which physically prevents the recruitment of co-activator or co-repressor proteins [32]. These pieces of evidence suggest a mechanism in which ligands activate COUP-TFII through the release of the receptor from its auto-inhibited state, even if a physiological ligand capable of inducing the activation of COUP-TFII has not yet been identified.

The absence of a classical ligand-binding pocket highlighted by the crystallographic analysis of the COUP-TFII structure reveals that this is probably a true orphan receptor [32]. Despite this, it is possible to claim that COUP-TFII is a ligand-regulated NR since its activity can be modulated by high concentrations of retinoid acids [32], as well as chemicals belonging to the naphthol family [33]; of interest is whereas retinoic acid activates COUP-TFII, synthetic naphthol compounds are inhibitory. Naphthol compounds are small polycyclic molecules characterized by the presence of the phenyl ring; they have been shown to interact with a small hydrophobic surface pocket in the LBD that is away from the putative interacting surface with co-activators. Surprisingly, the naphthol compounds do not inhibit the co-activators’ binding. Instead they determine a proteasome-independent degradation of the NR. Indeed, the naphthol analogues’ binding is believed to induce autophagy of COUP-TFII as evidenced by the formation of NR foci, especially in the cytosol [33].

## 3. Regulation of Cell Differentiation and Cell Function in Health

### 3.1. Mesenchymal/Stromal Cells Identity

Experimental pieces of evidence suggest that a time-specific and balanced expression of COUP-TFII is a general mechanism of regulation of cell differentiation as demonstrated, for example, by the role of the nuclear receptor in neurogenic-to-gliogenic stemness [44], in muscle or renal differentiation and in mesenchymal commitment and differentiation [20,45,46,47].

COUP-TFII is highly expressed in differentiating and adult mesenchymal cells and its expression influences cells terminal differentiation and fate choice, a function strongly associated to the regulation of stemness.

Hepatic and pancreatic stellate cells (HSC and PSC, respectively), as their names suggest, are cells residing in the liver and pancreatic parenchyma [48,49,50,51]. While HSC have been extensively investigated, studies on PSC are, in comparison, limited; nonetheless, HSC and PSC are substantially identical in function and the minimal differences in gene expression between them are probably due to the different micro-environment; hence, the knowledge on HSC functional role and differentiation are likely applicable to PSC. HSC are lipid accumulating cells located in the space of Disse and their main feature is the presence of vitamin A droplets. HSC, after cytokine stimulation, become “activated” and undergo a dramatic change of phenotype acquiring a myofibroblast-like phenotype and become pro-fibrogenic cells, expressing α-smooth muscle actin [52,53]. The transition between the quiescent and the activate state is apparently driven by COUP-TFII. The expression of PPARγ is necessary for lipid metabolism and for the preservation of the quiescent state; conversely, after activation by PDGF or TGF-β, HSC start expressing COUP-TFII while simultaneously reducing PPARγ expression. Interestingly, the alternative expression of COUP-TFII and PPARγ and the regulation of PPARγ activity (in the specific case of HSC, the suppression of its transcriptional activity) might be considered a general mechanism of cell specification for stromal cells (see below for bone stromal cells and adipocyte differentiation). Moreover, similar to endothelial cell differentiation, HSC COUP-TFII is downregulated by activation of the Notch pathway, suggesting that Notch-mediated inhibition of COUP-TFII might be another common mechanism of genetic regulation, which, together with Hedgehog (HH) signaling, may be important in cell fate decision and in sensing tissue integrity [54].

COUP-TFII is implicated in cell fate decision of bone marrow stromal cells (BMSC) and is necessary in the early phases of adipose differentiation of C3H10T1/2 cells and BMSC. Specifically, in BMSC, COUP-TFII is apparently pivotal for directing towards adipocyte, osteoblast, chondrocyte or myoblast differentiation, which are mutually exclusive specification routes. Molecular analysis performed to identify the drivers of these process show that COUP-TFII downregulates β-catenin and RUNX-dependent genes [55]. RUNX, a master regulator of bone development, may form dimers with COUP-TFII, and COUP-TFII/RUNX complexes are less efficient in activating RUNX-osteoclastic genes [56]. By blocking Wnt and RUNX2 and activating PPARγ, COUP-TFII induces pre-adipocytes commitment of BMSC whilst COUP-TFII-mediated activation of SOX9 allows the preferential development of chondrocytes and suppress pre-myoblast commitment [55]. Expression of COUP-TFII in osteoblast/adipocyte cell decision is under the control of miRNA 194 and fibroblast growth factor 2 (FGF2) [57,58]. miR-194 and FGF2 act in opposition: In the presence of osteogenic stimuli, miR-194 expression increases and, targeting COUP-TFII, induces osteogenic differentiation; conversely, the extracellular stimulation of FGF2 counteracts the effect of miR-194 increasing COUP-TFII expression through the MEK1/2 signaling pathway, further demonstrating its crucial role for the integration of signals determining cell differentiation.

The pivotal role of COUP-TFII in differentiation is also demonstrated by its interaction and reciprocal regulation with stemness factors, primarily OCT4. In undifferentiated cells, *COUP-TFII* transcription is actively repressed by OCT4. Interestingly, OCT4 allows the expression of the COUP-TFII-repressor miR-302. Hence, OCT4, directly and indirectly suppressing *COUP-TFII*, maintains the pluripotency of stem cells; conversely, when COUP-TFII expression and activity increases, such as during the initial commitment, *OCT4* is repressed. Repression of *OCT4* is COUP-TFII-dependent and requires the binding of COUP-TFII to the *OCT4* promoter. Interestingly, expression of COUP-TFII is self-sustained by a positive feedback loop, indicating that the expression of NR, once induced by still unknown mechanisms, inexorably leads to commitment and differentiation [41,59].

### 3.2. COUP-TFII in Metabolism and Metabolic Active Organs

COUP-TFII expression in metabolic tissues has been described since 2002 [60] and some of its functions and mechanisms of action has become clearer since then. COUP-TFII not only regulates energy expenditure in local tissues but it is also implicated in pancreatic insulin secretion and central nervous system response to available energy substrates.

#### 3.2.1. COUP-TFII, Energy Expenditure and the White Adipose Tissue

COUP-TFII expression is inversely associated to energy expenditure in metabolic organs. Heterozygous *COUP-TFII* +/− mice have a liner body, reduced gain of body weight, comparable brown adipose tissue (BAT) but reduced white adipose tissue (WAT) [61]. Interestingly, *COUP-TFII* +/− mice have increased glucose tolerance and glucose homeostasis that could be a consequence of increased energy expenditure and better glucose metabolism. The increased energy expenditure is probably associated to higher expression of uncoupling protein 1 (UCP1) and PPARγ co-activator 1α (PCG1α) and agree with the phenotype of COUP-TFII over-expressing mice in the heart that show a reduced metabolism, increased ROS production and altered mitochondria [17]. The reduced accumulation of WAT tissue points to a role of COUP-TFII in adipose differentiation. Indeed, silencing *COUP-TFII* in differentiating adipocytes reduces the accumulation of lipid droplets caused by a COUP-TFII-mediated inhibition of Wnt10 synthesis. Similarly, Okamura et al. [30] demonstrated that COUP-TFII is necessary for adipocyte differentiation: in 3T3-L1 preadipocyte cells, β-catenin induces the expression of COUP-TFII that then recruits the SMRT co-repressor to the *PPARγ* promoter, suppressing the expression of the master regulator of adipogenesis. The apparent discordance in the conclusions of the two papers might be explained by the observation that COUP-TFII expression increases in the initial phase of differentiation and precedes the expression of PPARγ, suggesting that COUP-TFII expression is necessary for the proliferation and commitment of pre-adipocytes [30]. Interestingly, by modulating PPARγ and adipocyte differentiation, COUP-TFII could influence the onset of obesity and diabetes [51].

The COUP-TFII-glucose link in metabolism is further confirmed by the finding that the overexpression of the NR increases lactate production in response to MEK activation. Lactate then activates the mTORC1 and further influences cell metabolism. Increased lactate production by lactate dehydrogenase activation is a hallmark of the Warburg effect and could be associated with an increased epithelial to mesenchymal transition (EMT) and with cancer progression [62].

#### 3.2.2. COUP-TFII and Liver Metabolism

In the liver, COUP-TFII is implicated in the control of fatty acid β-oxidation and glucose metabolism. The NR PPARα is the main regulator of fatty acid metabolism in the liver; it controls fatty acid β-oxidation and transport, and its expression is regulated by two NRs (HNF4α and COUP-TFII) that are caught in a regulatory loop. HNF4α and COUP-TFII receptors bind to the *PPARα* promoter with opposite effects on the same HNF4α-HRE: While HNF4α is a *PPARα* inducer, COUP-TFII binding to HNF4α-HRE reduces basal and HNF4α-induced *PPARα* expression, hence acting as a repressor [63]. Interestingly COUP-TFII expression is positively regulated by HNF4α (but the ability of the latter to induce COUP-TFII is reduced by high glucose concentrations) and PPARα binds to HNF4α-HRE, positively regulating its own expression [64].

COUP-TFII interacts with glucocorticoid receptor α (GR α) for the expression of CYP7A1, while the interaction of COUP-TFII with the GRα response elements repress the expression of GRα-activated genes by recruiting co-repressors [65]. Interestingly, the dimerization with GRα is necessary for the activation of the liver phosphoenolpyruvate kinase, the rate limiting enzyme of liver gluconeogenesis [65,66].

Bile acids are necessary for nutrients absorption but they also act as signaling molecules [67]; they stimulate cell metabolism and protect the liver from inflammation and steatosis, activating G-protein coupled receptors and FXR. As previously stated, CYP7A1 is the rate-limiting enzyme for bile acid synthesis from cholesterol and its expression is regulated directly by COUP-TFII, clearly suggesting that the NR may indirectly influence hepatic metabolism, regulating bile acid synthesis. Bile acids are the natural ligands of FXR that indirectly counteract the COUP-TFII-mediated activation of CYP7A1. Indeed, activated FXR increases the expression of the small heterodimer protein (SHP), a nuclear receptor belonging to the NR0 family, that sequesters LRH-1, an obligated factor for CYP7A1 expression [68].

In the liver and pancreas, glucose repression of COUP-TFII is modulated by insulin. Binding of insulin to its receptor activates the AKT pathway, which in the end brings forth the phosphorylation and nuclear exclusion of FOXO1 and *COUP-TFII* repression [69]. Moreover, hyperglycemia induces the ChREBP-mediated *COUP-TFII* downregulation, resulting in lipogenesis and glycolysis [69].

In newborn mice, *COUP-TFII* mRNA steadily increases and the *NR2F2* gene expression is enhanced directly by PPARα and indirectly, via cAMP, by glucagon. Newborn mice infected with a dominant negative COUP-TFII have impaired gluconeogenesis associated with a reduction in co-factors for fatty acid oxidation. Rescuing the PPARα activation with agonist Wy 14,643, Planchais et al. have hypothesized that COUP-TFII and PPARα co-operate for the regulation of β-oxidation and that COUP-TFII is necessary for the maintenance of correct glucose levels by gluconeogenesis in newborn mice [70].

The gene of the anabolic enzyme glucokinase is another COUP-TFII target; this kinase is induced by insulin and retinoids, and RARα, HNF4α and COUP-TFII cooperate to induce its expression [71].

#### 3.2.3. COUP-TFII and Insulin

**Figure 3:**
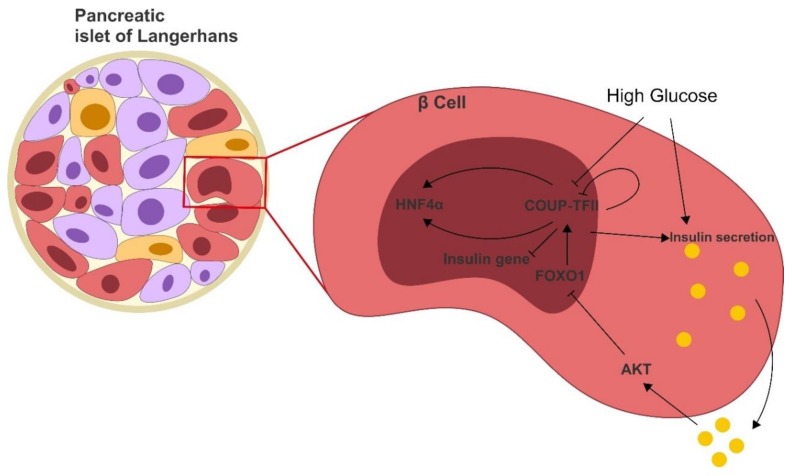
COUP-TFII and insulin in pancreatic β-cells. High glucose concentrations in the blood induce the release of insulin by β-cells and attenuate the expression of COUP-TFII. COUP-TFII is known to repress insulin genes, hence reducing the expression of COUP-TFII increases insulin gene expression but reduces insulin secretion. Interestingly, insulin in a paracrine manner further decreases *COUP-TFII* expression by nuclear exclusion of FOXO1 caused by AKT-mediated phosphorylation. HNF4α and COUP-TFII are reciprocally induced and sustain insulin secretion. Based on References [69,72,73,74].

COUP-TFII is a mediator of insulin secretion in pancreatic β-cells [72]. In pancreas, COUP-TFII is expressed in the endocrine compartment and β-cell-specific KO of *COUP-TFII* is lethal [73]. Interestingly, *COUP-TFII* +/− mice appear healthy but have a lower glucose tolerance associated with decreased insulin secretion after glucose stimulation, and they show a mild increase of insulin levels during fasting [73]. As previously cited, insulin represses COUP-TFII in peripheral tissues. Similarly, β-cells glucose-stimulated insulin secretion suppresses COUP-TFII in an autocrine manner trough a FOXO1-mediated mechanism, in an auto-regulatory feedback loop [69,74] (Figure 3).

Interestingly HFN4α (whose inactivation causes the maturity onset diabetes of the young 1 (MODY1) [75]) and COUP-TFII are reciprocally inducing each other whilst COUP-TFII auto-inhibits its expression in β-cells, providing a further layer of regulation for insulin secretion [64,74].

Besides the role of insulin regulation in β-cells, COUP-TFII is also important in their homeostasis; *COUP-TFII* KO in PDX1 expressing cells results in glucose intolerance and in β-cell reduction, essentially due to a reduced differentiation of progenitor cells. Mechanistically, β-cell homeostasis is maintained by a COUP-TFII-mediated increase of β-catenin expression and transcriptional activity. Moreover, induction of cyclin D1 and axin by glucagon-like peptide (GLP) (an intestinally produced hormone that induces insulin secretion in β-cells [76]) in *COUP-TFII* KO mice is reduced, suggesting that COUP-TFII is required for GLP β-catenin activation [77].

COUP-TFII is expressed at different intensities in the CNS areas (ranging from completely absent to strongly expressed [78,79]) that are believed to contribute to the patterning and function of the CNS [80]. Interestingly, COUP-TFII could also influence the CNS response to nutrients. Indeed, some of the neurons of the ventromedial hypothalamus, a region pivotal for the regulation of the energy balance, express the NR. Fed mice have higher COUP-TFII expression in the hypothalamus compared to fasted mice, and insulin, conversely to peripheral tissues, induces the expression of the receptor in this CNS area. Moreover, the fraction of ventromedial hypothalamic neurons expressing COUP-TFII are also positive for the steroidogenic factor 1 (SF1), a protein specifically expressed in neurons involved in energy sensing. In the ventromedial hypothalamus, SF1 is co-expressed with the melanochortin receptor, whose ligand is secreted in response to insulin. Interestingly, melanochortin receptors with the intermediation of cAMP induce *COUP-TFII*. The difference in insulin effects on COUP-TFII in the CNS compared to peripheral tissues are likely a consequence of insulin acting as an anabolic factor for the liver or the adipose tissue and catabolic in the central nervous system [81]. In agreement with this observation, *COUP-TFII* +/− mice in the ventromedial neurons display a liner phenotype, with increased energy expenditure, and are more prone to develop hypoglycemic-associated autonomic failure [82].

### 3.3. The Vein Identity and the Link to NOTCH

The observation that *COUP-TFII* KO mice show defects in angiogenesis, specifically in the appearance of venous and lymphatic vessels, prompted several groups to study the angiogenesis–COUP-TFII link [16].

Blood vessels arise early during embryogenesis to compensate for the increasing need of nutrients and oxygen of the growing and differentiating organs. Vasculogenesis, the de novo formation of blood vessels in embryo, is different from angiogenesis [83,84,85]. Whereas angiogenesis is the formation of blood vessels from existing ones, vasculogenesis is the process of differentiation of angioblasts as they coalescence to form the primitive vessel bed and is limited to the embryonic phase. After vasculogenesis, the first signs of arterial specification appear around day E8.5 with major morphological changes (e.g., the initial heart formation) between day E8 and E9. Venous and lymph markers appear later, usually after day E9–9.5 [85]. Despite the different morphology origin, vasculogenesis and angiogenesis are regulated by similar mechanisms with major players being the Notch signaling pathway and VEGF; other pathways involved are BMP/TGF-β and Hedgehog (HH) [84,85].

The Notch pathway is a cell-to-cell contact pathway that requires the presence of the membrane-bound Notch ligands Jagged and Dll in one cell, and the trans-membrane Notch receptors in the adjacent cells; the cells receiving the Jagged or Dll signal cleaves the Notch receptor, which ultimately determines the transcription of the Notch effectors Hes and Hey. Notch acts through lateral inhibition, a termed coined in neuroscience to describe the ability of an active neuron to reduce the firing of the neighbor cells. Notch lateral inhibition is a frequent phenomenon and it is fundamental for maintaining the intestinal architecture [86]. The steps necessary to activate the pathway and the underlying mechanisms have been extensively studied (for recent reviews see [86,87,88]).

During angiogenesis, vessel cells naturally develop towards the venous phenotype whereas Notch signals direct the cells toward the artery phenotype. Indeed, expression of the intracellular Notch receptor induces the expression of endothelial markers and arterial marker ephrin B2 and Nrp1 in normal differentiating cells and in endothelial cells after ischemia [89]. Conversely, KO mice for components of the Notch pathways die in utero due to defects in vessels morphogenesis [90,91]. In this process Notch appear pivotal in inducing cell differentiation but Notch expression is dispensable for cell proliferation.

Besides Notch, differentiating cells receive paracrine signals mainly by VEGF. Activation of VEGF Receptor 2 (VEGF-R2) by VEGF A and D induces the appearance of ephrin B and increases the expression of Dll-4. VEGF release in the extracellular matrix is controlled by HH paracrine secretion given that null mutants for HH do not express ephrin B2 [85]. The expression of Notch ligands in response to VEGF appears to be important during vessel sprouting and in the maintenance of the tip and stalk vessel growth. Tip cells express Dll-4 at high levels and induce the activation of Notch in the stalk cells, committing them to the arterial phenotype; at the same time, Notch reduces Dll and VEGF-R2 expression, suggesting that both pathways are strictly intertwined.

Starting from embryonic day 8, COUP-TFII expression in mice is detectable in the visceral mesoderm and differentiating heart [16]. General knock out of the receptor is lethal in utero (no mice are alive after E10) and it is associated with vast edema and hemorrhages in the brain and heart; a small percentage of haplo-insufficient mice show defects similar to what is observed in full KO mice, suggesting that even a partial reduction of the nuclear receptor is sufficient to induce the observed phenotype [14,16].

**Figure 4:**
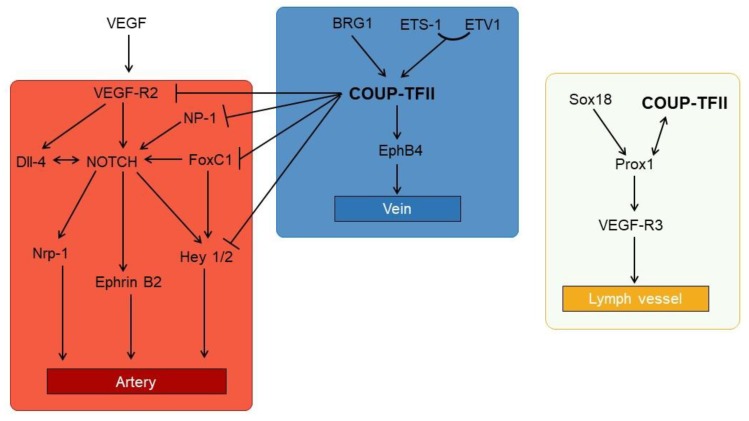
Cell signaling determination of artery, vein and lymph identity. COUP-TFII is one of the main determinants of vein specification. Early in differentiation, COUP-TFII, under the control of BRG-1 and ETS members (ETS-1 and ETV1), induces the expression of vein markers and represses components of the Notch pathway. Later, together with Sox18, COUP-TFII allow the expression of Prox1 and regulates lymphatic vessels differentiation. Based on References [84,85,92,93,94,95,96,97,98,99]. Redrawn from [85], published by Elsevier, 2019.

Expression of COUP-TFII induces vein genes expression (e.g., the receptor ephrb4) and later on lymphatic vessel specification (Figure 4).

COUP-TFII is related to both the VEGF and Notch pathway. Indeed, RIP-tag mice KO of COUP-TFII reduces insulinoma growth due to altered angiogenesis [18]; the observed phenotype is associated with a loss of repression of the decoy receptor VEGF-R1, an inhibitor of VEGF-R2 signaling. Moreover, COUP-TFII receptor controls angiopoietin expression in pericytes [92]. COUP-TFII blocks the Notch pathway, acting both upstream and downstream of Notch; upstream, COUP-TFII directly binds and reduces the expression of FoxC1 and NP-1, whereas downstream it binds to Hey2 [93]. The KO of *FoxC1*, a member of the Forkhead/Fox transcription factor family, reduces the expression of Dll-4 and NP-1, a co-receptor of VEGF-R2, and increases the expression of the lymphatic marker Lyve1. Moreover, KO of *FoxC1* and *2* reduces lymphatic sprouting due to lower VEGF-C expression [93]. Anyway, in the Notch pathway COUP-TFII is essentially a repressor of the transcription of artery-specific genes. In agreement with this hypothesis, endothelial cells with reduced COUP-TFII expression show alteration of G1/S progression, lower proliferation and higher expression of artery markers, all effects consistent with Notch activation; conversely, COUP-TFII in endothelial cells increases cell sprouting and cell proliferation, which is mediated by an increased expression of E2F1 that is regulated by both COUP-TFII and SP1 [100,101].

Endothelial progenitor cells (EPC) have high expression of vein markers (e.g., COUP-TFII) and low expression of arterial markers (e.g., Dll-4 and Hey2), at least in the vein patch [102,103]. The oxygen sensor HIF1α induces Dll-4 and Hey2 expression in EPC in hypoxia that leads to reduction of COUP-TFII expression [103]. Interestingly, Hey2 reduces HIF1α-mediated gene expression, creating a negative feedback loop and indicating that oxygen availability is another factor influencing the fate decision of endothelial cells modulating COUP-TFII and NOTCH expression [103].

Besides blocking Notch signaling, COUP-TFII actively induces vein and lymph node identity, regulating the expression of VEGF-C and D, as well as VEGF-R3 and NP-2 [94,95]. Expression of COUP-TFII in vein cells appear to depend on the transcription factor brahma-related gene 1 (BRG1) [96], a chromatin-remodeling factor whose presence in the promoter is required for COUP-TFII expression. Interestingly, BRG1 expression is also required for expression of Notch ligands [97,98]. Finally, COUP-TFII expression is directly regulated by E26 transformation specific 1 (ETS-1), a transcription factor belonging to the ETS family of transcription factor. ETS factors are involved in the regulation of angiogenesis, extracellular matrix remodeling and inflammation; they may also regulate cell metabolism and are involved in cancer progression [104,105,106]. ETS-1, along with the ETS factor ETV1, binds to the *COUP-TFII* promoter, increasing the NR expression, and suggesting that ETS angiogenesis is partially mediated by COUP-TFII [99].

Lymphatic vessel specification requires the expression of the transcription factor Prospero homeobox protein 1 (Prox1) that is detectable during development around day E9.5 in the cardinal vein [85]. Sustained expression of Prox1 is necessary to maintain lymphatic identity in the adult, and *Prox1* null mice do not develop lymphatic structures [107]. The transcription factor SRY-related gene Sox18 is expressed before Prox1 and is required but not sufficient for the Prox1 expression in developing embryos. Instead, COUP-TFII cooperates with Sox18 to induce *Prox1* expression in vein cells [108]. COUP-TFII directly binds to a conserved region within the *Prox1* promoter and it forms heterodimers with Prox1 [108,109]. Whereas COUP-TFII homodimers suppress the Notch pathway, Prox1/COUP-TFII dimers lack this ability resulting in a partial expression of Notch effectors in lymphatic vessels. Moreover, the heterodimers are permissive for the expression of lymph markers, such as VEGF-R3 (which binds VEGF-C), and they induce the expression of cyclin E1 [29,109,110,111]. In agreement with their role in lymphatic specification, COUP-TFII and Prox1 are suppressed by Hey1 and Hey2. Interestingly, the expression of Notch effectors transforms lymph cells in arterial-like cells, suggesting that the former are extremely plastic cells that, depending of the external stimuli, may give rise to all type of endothelial cells. This hypothesis is sustained by co-expression of all the three master regulators of endothelial specification in lymph cells (i.e., Notch, COUP-TFII and Prox1) [112].

## 4. COUP-TFII in Human Pathology

It is not surprisingly that COUP-TFII is linked to the development of several pathologies. The pathological role of COUP-TFII mirrors its function in health and is linked to, but not limited to, the regulation of cell metabolism, cell differentiation and, in cancer, to the regulation of the tumor micro-environment, especially angiogenesis.

### 4.1. COUP-TFII in Non-Cancer-Related Pathologies

#### 4.1.1. Cardiovascular Diseases

The overexpression of COUP-TFII, or the absence of its expression, is linked to the development of cardiovascular diseases, specifically arteriosclerosis and congenital heart defects (CHD). It is well known that arteries have a greater response to atherosclerosis factors compared to veins [113]; the reasons for such an epiphenomenon is not yet clear but could be caused by COUP-TFII. As documented before, endothelial cells of the vein and lymph vessels continue to express COUP-TFII. Interestingly, vein cells silenced for COUP-TFII and treated with angiotensin II have higher expression of atherogenic and inflammatory genes and downregulation of anti-thrombotic genes whilst the receptor overexpression has opposite effects [114,115]. The reduced susceptibility to atherosclerosis cannot be explained only on the basis of hemodynamic flow, which contribute to vascular remodeling [114,116]. Indeed, the available data suggest that COUP-TFII expression could protect the vein from atherosclerosis by a mechanism not yet clear but probably linked to the regulation of inflammation and lipid metabolism.

Deletions in chromosome 15q, the locus of *COUP-TFII*, are associated with abnormal facial appearance, in utero and postnatal growth retardations, as well as CHD. Mapping a 5.8 Mb deletion in a patient with CHD, Nakamura et al. [117] identified *COUP-TFII* as a putative candidate gene. Association of COUP-TFII to CHD was later confirmed by other groups [118,119,120,121,122]. Interestingly, deletion of 15q is not the only possible mutation happening in CHD in the *COUP-TFII* locus but single point missense mutations of the NR are sufficient to induce the pathogenic phenotype [120,122]. Recently, hyper-methylation of the promoter has been associated with the development of the double-outlet right ventricle [119], and a reduction in COUP-TFII expression, a consequence of polymorphisms or single point mutations of FOXC2, causes varicosity and valve failure in veins [123].

Although the evidence support the notion that COUP-TFII is necessary for the correct function and development of the cardiovascular system, and even suggest a possible protective action of the receptor, its overexpression may be deleterious, not only because it may be associated to the development of human malignancies but also because it may increase the risk of heart diseases. In mice, selective overexpression of COUP-TFII in the myocardium is a causative agent of dilated cardiomyopathy followed by heart failure and increased death rate. As previously stated, COUP-TFII is an important regulator of cell metabolism and alteration of cellular metabolism have been suggested as causative agents of heart failure [17]. Overexpression of COUP-TFII impairs substrate utilization of fatty acids and a general reduction of glucose usage, due to altered expression of the glucose transporter Glut-4, the hexokinase 2 and the 6-phosphofructokinase; moreover PGC-1a, a target of COUP-TFII in adipocytes differentiation, is downregulated in cardiomyopathy [17,61,124]. Finally, the metabolic changes are accompanied by increased ROS production, lower ATP output and mitochondria dysfunction due to a direct inhibition of genes regulating mitochondrial dynamics [17]. Consistent with the regulation of glucose utilization, COUP-TFII expression is regulated by glucose availability in endothelial cells: Acute administration of glucose increases COUP-TFII independently from insulin, whereas chronic administration reduces it. Finally, the silencing of the nuclear receptor in HUVEC cells increases eNOS and glucose transporters, suggesting a time- and glucose-dependent regulation of the receptor that could be important in the context of diabetes [125].

#### 4.1.2. COUP-TFII and Diabetes

The association of COUP-TFII and diabetes may be deduced by its role in insulin and glucose homeostasis and by its role in adipocyte differentiation, which we have already discussed. Specifically, the interaction of COUP-TFII with PPARγ and β-catenin could have a potential impact in the regulation of oxidative stress, inflammation and obesity development, which are linked to or are causative agents of diabetes [51,126]. Noteworthy, the association with diabetes is confirmed by data collected in the clinical study “Epidemiologic study on the insulin resistance syndrome (DESIR)” [127]. Subjects with the COUP-TFII polymorphism SNP-rs3743462 have lower blood insulin concentrations due to reduced COUP-TFII expression, similarly to what was observed in *COUP-TFII* +/− mice. This polymorphism is in a distal conserved region of the COUP-TFII promoter; mechanistically, it determines an increase binding of COUP-TFII that results in auto-repression of the nuclear receptor and altered insulin secretion and production [64].

In agreement with its function in vessel identity specification, increased COUP-TFII expression, paralleled by modifications of SOX18 and Prox1, is associated to brain arteriovenous malformations (AVM) in humans [128,129]. Anatomically, AVM are a net of dysplastic blood vessels without capillaries between the venous and arterial compartments. Immunohistochemistry analysis of these vessels show the contemporary expression of arterial and venous markers in the lesions with an increased expression of endoglin, indicating that the vessels are not properly differentiated [129]. Endoglin is a TGF-β co-receptor necessary for angiogenesis [130] and its haplo-insufficiency is associated with hereditary hemorrhagic Telangiectasia, a hereditary form of vessel malformation; null mice for the TGF-β co-receptor are defective in vessel differentiation and show ectopic COUP-TFII expression in the arterial compartment [130], a piece of evidence that strengthen the notion of COUP-TFII as a causative agent of AVM. Finally, a 15bp genetic deletion of the hinge region, necessary for the interaction with a friend of GATA2 (FOG2), reduces systolic and diastolic pressure and suggest that the NR is a determinant of essential hypertension in human [131].

#### 4.1.3. Infertility and Alterations of Mesenchymal Commitment

Alteration of COUP-TFII expression in the stromal compartment is associated with infertility and alteration of mesenchymal stem cell commitment.

Female and, possibly, male human infertility are linked to COUP-TFII expression [132]. COUP-TFII is a marker of mesenchymal Leydig stem-like cells [133] and it is a direct regulator of INSL3 hormone produced by Leydig cells [134]. Moreover, COUP-TFII indirectly regulates testosterone levels influencing the expression of genes involved in testosterone biosynthesis [132,135]. INSL3 determines testicular descent and has been linked to oocyte maturation and male germ survival in adults; its expression is reduced in human pathologies [136,137]. Pre-puberal COUP-TFII deletion causes hypogonadism and infertility, due to almost absent spermatozoa [132]; these effects were largely associated with a reduction of mature Leydig cells and are consistent with a lower testosterone production but also might be explained by reduced INSL3 production. Interestingly, COUP-TFII deletion in adult mice has no effect on testosterone levels and Leydig cells appear normal in number, suggesting that the receptor is dispensable for Leydig function in adults [132]. Recently, a 3 Mb deletion of the locus containing NR2F2 was described in an SRY 46, XX male with spontaneous male puberty, further implicating COUP-TFII in sex determination [138].

In female, the occurrence of endometriosis is often a cause of infertility. Endometriotic tissues exclusively express the aromatase P450 that in eutopic endometrial stromal cells is repressed by COUP-TFII; aberrant expression of P450 increases estrogen production that, in turn, induces the expression of prostaglandin and inflammatory cytokines favoring endometriosis progression [139]. In endometriotic tissues, the P450 COUP-TFII-mediated inhibition is counteracted by SF1 that binds to the same promoter region [140]. In humans, the expression of COUP-TFII is often reduced in endometriosis compared to healthy tissues [141]. A possible reason for this reduction is the SF1 indirect regulation of COUP-TFII. Conversely, to COUP-TFII, SF1 is silenced by methylation in eutopic endometrium but is highly expressed in endometriosis and hence it might repress COUP-TFII allowing the expression of P450 [142]. Indeed, Expression of SF1 is sufficient to promote the formation of enlarged glands and to alter the progesterone–HH–COUP–TFII pathway [142].

In endometrial tissues COUP-TFII regulates angiogenesis, adhesion and inflammation [141]. The inflammatory cytokines IL-1β, TNF-α and TGF-β reduce COUP-TFII by means of the intermediary miR-302a that targets the 3′-UTR of the NR mRNA; a consequence of the COUP-TFII downregulation is the activation of COX2 [143] and the hypoxia-dependent expression of angiopoietin [144], two factors that could promote inflammation and angiogenesis.

In uterine stroma COUP-TFII is necessary for proper implantation and decidualization. COUP-TFII expression is regulated by the progesterone–Indian HH axis deriving from epithelial cells. Mesenchymal cells respond to Indian HH producing BMP that causes decidualization in vivo [145]. Inactivation of the NR in uterine mesenchymal cells leads to miscarriages during mouse pregnancy caused by improper placental vascularization [146]. Interestingly, COUP-TFII silencing in human primary endometrial stromal cells is not sufficient to completely abrogate decidualization but nonetheless alters genes involved in inflammation, angiogenesis and cell adhesion [141].

As previously discussed, quiescent HSC are substantially COUP-TFII negative but when activated they start expressing COUP-TFII and losing PPARγ. Sustained activation of HSC brings forth excessive and uncontrolled deposition of the extracellular matrix that is associated with the development of liver cirrhosis [54]; similarly, activation of PSC is believed to be the main cause of the desmoplastic reaction of pancreatic ductal adenocarcinoma (PDAC) [50]. COUP-TFII is highly expressed in cirrhotic liver and its expression modulates genes involved in wound healing, specifically in angiogenesis, immunoresponse and extracellular matrix remodeling [54]. Interestingly, angiogenesis is usually linked to hypoxia, but HSC COUP-TFII-mediated angiogenesis is hypoxia and HIF-1α independent, but NF-κB dependent, suggesting that COUP-TFII-mediated angiogenesis may be important in the early phases of the healing response [54].

Finally, COUP-TFII has been also associated with the formation of congenital diaphragmatic hernia (CDH) and stomach malformations. CHD is a lethal anomaly that occurs in childhood, which can be caused by partial deletion of chromosome 15q [147]; consistent with this observation, *COUP-TFII* KO mice show diaphragmatic defects similar to the Bochdalek-type hernia phenotype and a frameshift mutation of NR2F2 was recently identified in a patient with CHD [147,148].

COUP-TFII and HH are important determinants of gastric differentiation. Deletions of components of the HH pathway have been associated with congenital gastrointestinal defects, and loss of HH expression causes intestinal metaplasia of the stomach [149,150,151]. Moreover, *COUP-TFII* null mice in gastric mesenchymal cells have defects in radial patterning and show epithelial outgrowth; interestingly, these defects may be recapitulated by treating mice with the HH inhibitor cyclopamine, suggesting that COUP-TFII is not only a HH target gene but is also essential for HH action in the stomach [152,153]. Mechanistically, HH produced by epithelial cells induces the expression of COUP-TFII in mesenchymal cells to block the excessive expansion of circular smooth muscle cells and enteric neurons. At the same time COUP-TFII indirectly inhibits the epithelium proliferation; the mediator of this negative feedback loop is probably the secreted factor BMP-4, as demonstrated by the fact that the reduction of BMP signaling increases epithelial proliferation [154,155].

### 4.2. COUP-TFII in Cancer: The Good Guy and the Bad Guy

The association of COUP-TFII to cancer progression has been widely investigated and it has received more attention since the demonstration that the NR is a major player in cell differentiation and cell metabolism. Expression of COUP-TFII and other nuclear receptors (e.g., LXRβ, RARα, REVERBα) is relatively high in cancer cells tested by high-throughput qPCR; moreover, the receptor forms a cluster with RXRα, HNF4 (α and β) and PPARγ, reflecting known functional associations and is relatively near to ERα, who is implicated in breast cancer progression (see below for details) [138]. Interestingly, COUP-TFII negatively correlates with the resistance to some chemotherapy agents, especially to derivatives of microtubule-targeting drugs (e.g., Taxol or colchicine), pointing to a putative association with a cytoskeleton organization and EMT [156].

Nonetheless, reflecting the number of putative and demonstrated functions, as well as the ever increasing interactome of COUP-TFII, it is not entirely surprising that a consensus is not reached on its function and we may differentiate among tumors where COUP-TFII might act as onco-suppressor or as oncogene, with some gray areas in-between (Table 1).

#### 4.2.1. The Gastrointestinal Cancers: Mostly an Oncogene

In the gastrointestinal tract (i.e., intestine, pancreas and stomach) COUP-TFII acts both as an oncogene and as an onco-suppressor.

Despite the in vitro and in vivo results on animal models, there is no consensus on COUP-TFII in primary colorectal cancer, and there are probably too few data to reach a meaningful conclusion. In 2009, Shin et al. [157] reported that COUP-TFII-expressing Korean patients have increased overall survival compared to non-expressing individuals, although borderline significant. The same group confirmed these results in 2017: Studying LXR, they found out that its expression and the absence of COUP-TFII were both negative prognostic factors and were associated to shorter overall survival [158]. However, two different groups reported that high expressing COUP-TFII patients, compared to low expressing patients, have increased risk of metastasis and lower overall survival [159,160]. Interestingly, in primary tumors COUP-TFII correlates with SNAIL1 and SMAD4 and in cell lines with the loss of SMAD7 [159,160,161]. SNAIL1 is a transcription factor usually expressed during EMT; its expression has been demonstrated to induces EMT in gastric cancer [183] although it may direct the cells towards a low proliferating phenotype trough the β-catenin-LEF1 complex [184]. SMAD7 instead is the only inhibitor member of the SMAD proteins that mediate the action of EMT-inducers TGF-β and BMP [185,186,187]. These observations agree with in vitro experiments and animal models of colorectal cancer, where COUP-TFII facilitates cell invasion and EMT [159,161,188]. *COUP-TFII −/−* mice in the colon mucosa, treated with DMH/DSS to induce colon cancers, have lower liver metastasis compared to wild-type mice. This effect was further investigated in vitro in human cancer cells where TGF-β-mediated EMT was reduced (as demonstrated by an increase in ZO, membrane β-catenin and E-cadherin) after silencing the NR: The culprit was found in the reduction of SNAIL1, whose expression is directly regulated by COUP-TFII [159]. Apparently, COUP-TFII is at the center of a miRNA network that elegantly explain the loss of EMT. MiR-382 is an onco-suppressor in non-small-cell lung, ovarian, prostate and esophageal cancers [189,190,191,192], and it is downregulated in colorectal cancer where it inhibits tumor metastasis, blocking SP1, and sensitizes cancer cells to chemotherapy [193,194]. Interestingly, COUP-TFII potentiates the action of SP1 in angiogenesis and development [92,94], recognizing SP1 response elements and miR-382 directly binds COUP-TFII 3′-UTR, knocking down the receptor, and hence downregulates two transcription factors that are demonstrated to synergize and induce EMT. Besides being regulated by miRNAs, COUP-TFII is also a miRNA-regulatory factor, specifically of miR-21 and miR-34a in colon cancer [161,162]. MiR-21 inhibits the phosphatase and tensin homologue (PTEN) and programmed cell four, and it is up-regulated in cancer compared to adjacent tissues; its circulating levels have been proposed as a potential biomarker in colorectal cancer, together with other miRNAs. Moreover, miR-21 might increase prostaglandin E2 levels (a potent mediator of inflammation), inhibiting 15-hydroxyprostaglandin dehydrogenase. MiR-34a is a tumor suppressor miRNA: In colorectal cancer its expression is usually downregulated, often because of TGF-β action or long non-coding RNA acting as sponge for the miRNA [195,196]. Key features of miR-34a are the inhibition of cell migration and its chemosensitizer role in colorectal cancer [197,198]. COUP-TFII directly induces the expression of miR-21 in TGF-β-treated cells, resulting in the downregulation of SMAD7 whereas it reduces miR-34a [161,162]; hence, modulating miR-21a and miR-34a, COUP-TFII strengthen its action as tumor oncogene and induces EMT in colorectal cancer.

Although COUP-TFII expression in pancreatic tissue was reported long ago [199], its implication in PDAC has only recently been demonstrated [163,199]. In pancreatic cancer patients COUP-TFII correlates with a worse prognosis and lower survival; in vitro and in vivo experiments revealed that COUP-TFII promotes invasiveness and anchorage-independent growth of PDAC cells, and it plays a fundamental role in neoangiogenesis by directly modulating VEGF-C [163].

There are limited and conflicting data on COUP-TFII in gastric cancer. Feng et al. [166] have hypothesized that COUP-TFII might be an oncogene given its significant up-regulation in primary cancers compared to normal tissues and the overall shorter survival of patients with elevated COUP-TFII expression. However, different pieces of evidence suggest the opposite. Indeed, downregulation of COUP-TII and up-regulation of HNF4α has been linked to the development of gastric intestinal metaplasia, a frequent precancerous lesion of the stomach, indicating a protective role of the NR in gastric cancer progression [164]. In line with this finding, COUP-TFII is more expressed in normal gastric mucosa cells (GES-1) compared with gastric cancer cells and gastric carcinoma tissues. Moreover, GES-1 cells with *COUP-TFII* knocked down have increased proliferation and invasiveness while COUP-TFII overexpression in xenograft tumor models reduces the tumor burden [165]. Finally, confronting the pathological data from 80 gastric cancer patients obtained from the Oncomine database, Ding et al. demonstrated that high expression of COUP-TFII correlates with increased survival [165], in stark contrast with the results of Feng et al. [166]. It is conceivable that this discrepancy arises from the use of different databases, the use of different selection criteria of the study population and the probe tested. Given the in vitro and in vivo results we may hypothesize that COUP-TFII is probably an onco-suppressor in the gastric cancer, but more experiments need to be done to fully understand its action in this tumor.

#### 4.2.2. The Uncertainty of COUP-TFII in the Breast Cancer

Expression of COUP-TFII in primary breast cancer has been reported in 47% to 59% of cases analyzed by immunohistochemistry [95,175]. Whereas an initial report suggested a positive correlation between COUP-TFII and tumor progression, specifically with a worse outcome and lymph node metastasis [95] (who were linked to VEGF-C expression), recent evidence have challenged this idea, suggesting that COUP-TFII may play different roles during breast cancer progression [175,176,177,178,179]. The expression of the estrogen receptor α (ERα) is one of the main discriminants in the choice of breast chemotherapy. Interestingly, COUP-TFII expression is positively associated with ERα expression and it is reduced in endocrine-resistant cell lines, independently of ERα expression. Indeed, tamoxifen resistant (TAM-R) MCF-7-derived cells are ERα positive but low-expressing COUP-TFII cells and re-establishing COUP-TFII expression in TAM-R have no effects per se but enhances the antiproliferative response to 4-OHT. Interestingly, reducing the expression of COUP-TFII in MCF-7 cells transforms 4-OHT from an antagonist to an agonist of cell proliferation. These phenotype changes are also reflected in the expression of progesterone receptor (PR) and pS2, two known targets of ERα, suggesting that the COUP-TFII role is more complex and the final response to its expression could arise from integrating different stimuli.

ERα controls COUP-TFII expression, given that mRNA of the latter increases after either overexpression of ERα or treatment with E2, but not in TAM-R cells [175]. Nonetheless, regulation of COUP-TFII expression is only partially associated with an ERα direct transcription activity and requires the presence of a functional MAPKs cascade [200]; hence, it could be dependent on the presence of growth factors or to a transcription-independent ERα activity, both resulting in activation of MAPK. Using genomic localization maps, Erdos and Balint [201] have recently mapped the COUP-TFII cistrone, demonstrating its co-localization with master transcription factors, namely ERα in MCF-7 cells, HNF4 in HepG2 and GATA-binding factors in K562 cells [201]. Co-presence of ERα and COUP-TFII marks the promoter area as an active enhancer and suggests that the orphan receptor might mediated the effects of the hormone. Similar results were reached by a different group almost at the same time, highlighting the importance of COUP-TFII in mediating ERα transcription activity, and extending the interaction of COUP-TFII to pioneer factors FoxA1 and GATA3 whose co-presence is apparently necessary for creating a strong enhancer region accessible to ERα [177].

Several pieces of evidence suggest that COUP-TFII could partially act like a tumor-suppressor. First, its expression increases p21/WAF and cyclin D1 delaying the transition trough the G2/M phase [180]; second, COUP-TFII interacts with nucleolin and it is required for the expression of RARB2, a known tumor suppressor [175]; lastly, these results are confirmed by the observation that COUP-TFII is negatively associated with EMT transition induced by TGF-β, and to the resistance to chemotherapy and to changes in E-cadherin and Slug, the latter two being markers of EMT [179]. Downregulation of the NR in tamoxifen-resistant cells is probably linked to epigenetic modifications; treatment with 5-aza-2′-deoxycitidine (AZA), a demethylating agent, or with the deacetylase inhibitor trichostatin, increases COUP-TFII expression in TAM-R cells [176]. Among the 7 GpC islands located in the *NR2F2*, the most methylated is located on the first exon. The increased methylation observed in vitro is also confirmed in human breast tumors compared to healthy breasts.

The acquisition of hormone resistance has been associated to an increased expression of NF-κB, a known modulator of inflammation, redox and metabolic status, and competitively associated with several nuclear receptors [126]. As in other organs and pathologies, COUP-TFII inhibits NF-κB activation and down-targets expression, co-immunoprecipitating with RelB and NF-κB in Mcf-7 cells. Furthermore, COUP-TFII and NF-κB status are inversely correlated in patients, and their association has been suggested as a possible explanation of acquired hormone resistance following COUP-TFII downregulation [181].

An interesting feature of breast cancer progression is vascular trans-differentiation, or vascular mimicry, i.e., the ability of epithelial tumor cells to convert into endothelial-like cells, which initially facilitates the local tumor growth and later its spreading [202]. In breast cancer, the expression of endothelial markers in tumor cells can be induced by retinoic acid that noteworthy reduces cell proliferation. Interestingly RA-treated cells show activation of COUP-TFII and *COUP-TFII* KO prevents the formation of vessel-like structure in Matrigel but has no effect on VE-cadherin expression and does not influence cell fusion. Hence, apparently, although COUP-TFII is expressed and required for a specific step of vascular mimicry, it is conceivable that it could mediate the RA-induced reduction of cell proliferation [182].

#### 4.2.3. Prostate and Renal Cancers

Recent studies revealed that in prostate cancer (PC) and in renal cell carcinoma (RCC) COUP-TFII is a putative oncogene [167,168,169,170,171,172,173,174,203].

COUP-TFII expression in primary PC is greater than in healthy prostate epithelium, with the NR amount correlating to an increased metastatic potential and reduced recurrence-free survival [167,168,169]. However, overexpression of COUP-TFII is not sufficient to induce tumor development but it potentiates the metastatic capacity in the indolent *PTEN −/−* mouse model of PC [169]. In these mice the loss of *PTEN* is usually accompanied by the activation of the TGF-β pathway, which induces the growth of an extracellular barrier that restricts prostate cancer growth and progression [169,203]. While TGF-β apoptotic and cytostatic signals are counteracted by the activation of AKT and mTOR in the *PTEN −/−* background, to become metastatic PC cells requires the overexpression of COUP-TFII. Indeed, in vivo experiments show that NR overexpression in *PTEN −/−* prostatic epithelium leads to increased cancer cell proliferation and invasiveness; these effects are a consequence of the direct binding and sequestration of SMAD4 by COUP-TFII that causes the loss of the TGF-β signal and the reduction of TGF-β-induced growth barrier [169]. Furthermore, the association of the NR with metastasization is also underscored by the finding that COUP-TFII expression positively correlates with p-mTOR expression and both are predictors of increased lymphangiogenesis and lymph node metastasis in PC primary samples [170]. The link with mTOR points to a role of COUP-TFII in the regulation of PC metabolism. Indeed, the increased expression of COUP-TFII in PC cells induces a reduction of the mitochondrial pyruvate carrier 1 (MPC1) expression. MPC1 is a regulator of cancer cells metabolism, which together with MCP2 forms a complex capable of regulating pyruvate transport within mitochondria [172]; the reduction of MPC1 mediated by COUP-TFII has a negative impact on mitochondrial pyruvate import and induces the cells to use different intermediate substrates for the TCA cycle and at the same time increases glycolysis, promoting tumor growth and invasion of prostate cancer cells [172]. Finally, COUP-TFII is repressed by miR-382, 101 and 27a, whose expression is often reduced in PC [171,192]. The knock-down of these miRNAs increases PC proliferation and invasiveness; mechanistically, it allows the expression of EMT markers SNAIL and matrix metalloproteinase 2 and increases cell proliferation and invasiveness in a COUP-TFII-dependent manner. Moreover, miR-101 and miR-27a negatively regulate the resistance to the chemotherapy drug enzalutamide by indirectly downregulating the expression of the COUP-TFII down-targets FoxM1 and centromere protein F (CENPF) [171].

In RCC, the most common type of kidney cancer, COUP-TFII expression is considerably higher in cancer tissues and cancer cell lines than in healthy counterparts. Moreover, elevated levels of COUP-TFII correlate with a poor prognosis and are an indicator of poor survival [173,174]. The COUP-TFII oncogenic role in RCC may be due to the regulation of the cell cycle and apoptosis. In fact, in vitro and in vivo experiments have shown that COUP-TFII silencing inhibits cancer cell proliferation, reducing the number of cells in the S-phase and inducing cell apoptosis, increasing the expression of pro-apoptotic factors, including cytochrome C, BCL2 Associated X (BAX) and BRCA1 [173,174].

## 5. Conclusions

The orphan nuclear receptor COUP-TFII is clearly implicated in embryogenesis and organogenesis as demonstrated by the lethal KO phenotypes [16]. In adults its expression is widespread with peak expression in cells of mesenchymal origin. Accumulating pieces of evidence show that it is implicated in tissue homeostasis and maintenance and it is a major regulator of cell differentiation and angiogenesis [16,20,46,54,55].

The importance of COUP-TFII in endothelial identity is well documented; the choice of arterial versus venous fate is a result of a cross-talk between Notch and COUP-TFII signaling, while the interactions of Brg1, Prox1 and COUP-TFII are central for lymphatic development [96,100,111,204]. The ability to direct angiogenesis is paralleled by its role in mesenchymal cell differentiation and point to a role of the NR in regulating the cells micro-environment with evident implications for several pathologies [45,54,74,95,117,128,132,146]. Indeed, the NR expression (or the absence thereof) is associated with the unfavorable progression of malignancies in several organs [157,163,165,167,173,181]; besides, COUP-TFII is linked to pathologies considered cancer risk factors, such as diabetes or cirrhosis [54,64].

Association with diabetes point to its role in the metabolic machinery: COUP-TFII is a regulator of adipocyte differentiation and lipid metabolism interacting or regulating other transcription factors, mainly of the PPAR family. Moreover COUP-TFII is implicated in glucose homeostasis in metabolically active tissues where increased expression of the receptor leads to reduced metabolism, higher ROS production and mitochondria disfunctions [17].

Still, there are several aspects of COUP-TFII physiology that need to be addressed by future researches.

First, it is apparent that COUP-TFII action is often context dependent, hence the identification of COUP-TFII partners, and the mechanisms of interactions with them could help shed light on some of the more complicated and difficult-to-explain results (e.g., the role in adipogenesis or the different roles of COUP-TFII in breast cancer).

Second, for historical reasons the research on COUP-TFII is essentially focused on the role of the canonical isoform, but new isoforms of COUP-TFII have been recently discovered. The lack of the DBD in these isoforms suggest that they might act with still unknown mechanisms. Interestingly, they also could be a different gene, or at least produced by an alternative promoter.

Third and finally, the discovery of specific and safe agonists and antagonists is mandatory for translational research. Even if COUP-TFII could easily be a true orphan receptor, the idea of COUP-TFII modulation for treatment is nonetheless absolutely of interest, although it is currently hampered by the lack of specific ligands. Several steps forward have been recently made with the discovery that naphthol compounds may modulate the NR, essentially inducing its degradation, but the research of COUP-TFII ligands is still in its infancy. Although the inhibition of COUP-TFII could be of interest for the treatment of some cancers or pathologies like cardiomyopathies, other pathologies, like infertility, could get therapeutic benefits from the use of COUP-TFII agonists.

## Figures and Tables

**Table 1:** COUP-TFII in cancer: role and molecular effects.

Cancer	Role	Ref.	Effects
Colorectal cancer	Onco-suppressor (?)	[157,158]	↑survival rate
	Oncogene (?)	[159,160,161,162]	↑risk of metastasis
			↓overall survival
			↑invasion
			↑EMT
Pancreatic cancer	Oncogene	[163]	↓survival rate
			↑invasiveness
			↑anchorage-independent cells growth
			↑neoangiogenesis
Gastric cancer	Onco-suppressor (?)	[164,165]	↓proliferation
			↓migration
			↓invasiveness
			↓cell growth
			↓metastasis
			↑survival rate
	Oncogene (?)	[166]	negative prognostic role
Prostate cancer	Oncogene	[167,168,169,170,171,172]	↑tumor cell growth
			↑proliferation
			↑metastatic potential
			↑invasion
Renal cancer	Oncogene	[173,174]	↓survival rate
			↑proliferation
			↓apoptosis
Breast cancer	Oncogene(?)	[95]	↓survival rate
			↑lymph node metastasis
	Onco-suppressor(?)	[175,176,177,178,179,180,181,182]	↑effects of Tamoxifen (TAM)
			↓EMT
			↓proliferation
